# New Insights into the Use of Empagliflozin—A Comprehensive Review

**DOI:** 10.3390/biomedicines10123294

**Published:** 2022-12-19

**Authors:** Joanna Forycka, Joanna Hajdys, Julia Krzemińska, Piotr Wilczopolski, Magdalena Wronka, Ewelina Młynarska, Jacek Rysz, Beata Franczyk

**Affiliations:** 1 Department of Nephrocardiology, Medical University of Lodz, ul. Zeromskiego 113, 90-549 Lodz, Poland; 2 Department of Nephrology, Hypertension and Family Medicine, Medical University of Lodz, ul. Zeromskiego 113, 90-549 Lodz, Poland

**Keywords:** empagliflozin, type 2 diabetes mellitus (T2DM), heart failure (HF), chronic kidney disease (CKD)

## Abstract

Empagliflozin is a relatively new drug that, as an inhibitor of the sodium–glucose cotransporter 2 (SGLT2), causes increased urinary glucose excretion and thus contributes to improved glycemic control, better glucose metabolism, reduced glucotoxicity and insulin resistance. Although its original use was to induce a hypoglycemic effect in patients with type 2 diabetes mellitus (T2DM), empagliflozin has also shown a number of other beneficial effects by demonstrating a nephroprotective effect, and it has proven to be a breakthrough in the treatment of heart failure (HF). Empagliflozin has been shown to reduce hospitalizations for HF and the number of deaths from cardiovascular causes. Empagliflozin treatment also reduces the incidence of renal events, including death from renal causes, as well as the risk of end-stage renal failure. Empagliflozin appears to be a fairly well-tolerated and safe drug. In patients with inadequate glycemic control, empagliflozin used in monotherapy or as an adjunct to therapy effectively lowers fasting blood glucose, postprandial blood glucose, average daily glucose levels, glycated hemoglobin A_1C_ (HbA_1C_) and also leads to significant weight reduction in patients with T2DM. Unfortunately, there are some limitations, e.g., severe hypersensitivity reaction to the drug and a glomerular filtration rate (GFR) < 30 mL/min/1.73 m^2^. As with any drug, empagliflozin is also characterized by several side effects among which symptomatic hypotension, troublesome genital fungal infections, urinary tract infections and rare ketoacidosis are characteristic.

## 1. Introduction

Empagliflozin is an oral glucose-lowering drug that was approved and came into clinical use in 2014 [1]. It belongs to the sodium-glucose cotransporter 2 (SGLT2) inhibitor group [2,3,4]—medications introduced for the treatment of the type 2 diabetes mellitus (T2DM). SGLT2 is the main transporter responsible for the reabsorption of glucose from the glomerular filtrate and represents a novel target in the treatment of T2DM [5]. By potently blocking glucose reabsorption in the proximal tubule, it increases urinary glucose excretion [2,6,7,8]. Among the currently used or tested SGLT2 inhibitors, empagliflozin has the highest selectivity for SGLT2 over sodium–glucose cotransporter 1 (SGLT1) (2500-fold) [9].

Chemically, empagliflozin belongs to C-glucosides [2,7], which determines the greater metabolic stability of the drug compared to O-glucoside derivatives. In the case of the O-glucoside derivatives, hydrolysis by intestinal enzymes occurs, resulting in low absorption [2]. C-glucoside derivatives have a favorable pharmacokinetic and pharmacodynamic profile. They only need to be used once daily. Moreover, renal or hepatic impairment does not significantly affect the concentration of the drug (or its metabolites) in the body [2,7].

**Figure 1:**
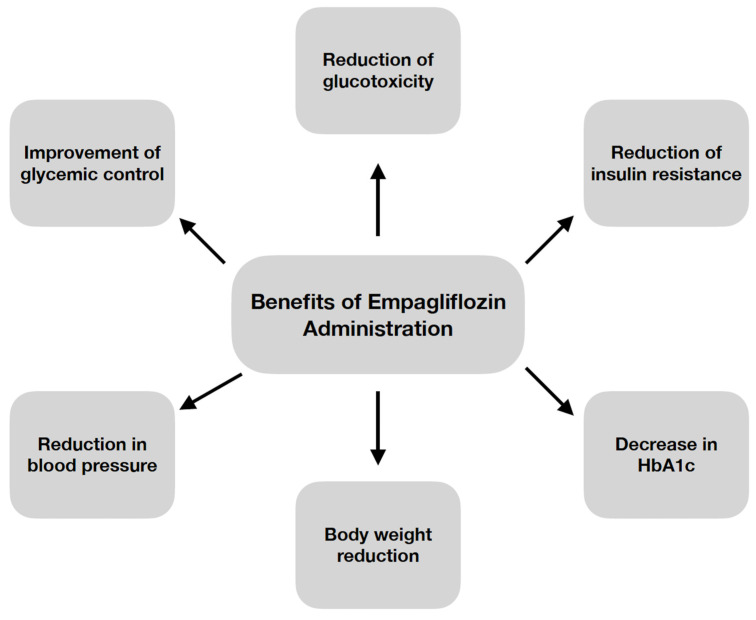
Beneficial effects of empagliflozin administration [2,3,4,6,7]. HbA1c, glycated hemoglobin A1C.

Empagliflozin acts selectively on SGLT2 transporters [2,3,7,8] compared to SGLT1 [3]. This is essential due to the fact that SGLT2 is expressed in proximal tubule segments [2,4,6], where 90% of glucose reabsorption occurs [2]. On the contrary, SGLT1 can be expressed in the intestines, heart, skeletal muscle [4], as well as in the proximal tubule segment responsible for only 10% of glucose reabsorption [2]. In addition, it has been shown that SGLT1 inhibition can lead to malabsorption, resulting in diarrhea [3]. The effects of empagliflozin result in a lowering of the renal threshold for glucose [2]. This leads to glucosuria and a decrease in plasma glucose levels [2,4,6,7] as well as a reduction in glycemic spikes and glycated hemoglobin A_1C_ (HbA_1C_) levels (with prolonged use) [7]. Empagliflozin has pleiotropic effects. Some of its beneficial effects are shown in Figure 1 [2,3,4,6,7]. Moreover, empagliflozin has been demonstrated to reduce oxidative stress. The administration of this medication causes inhibition of reactive oxygen species (ROS) production, reduces the activity of prooxidant factors, as well as improves mitochondrial function [10].

In T2DM, the starting dose is 10 mg for monotherapy as well as in add-on combination therapy administered orally. Since the mechanism of SGLT2 inhibition is independent of circulating insulin levels or insulin sensitivity, these agents can be combined with all other antidiabetic classes, including exogenous insulin. When used with a sulfonylurea or with insulin, a lower dose of the sulfonylurea or insulin may be considered to reduce the risk of hypoglycemia. For patients tolerating empagliflozin 10 mg once a day who have an estimated glomerular filtration rate (eGFR) ≥ 60 mL/min/1.73 m^2^ and are in need of better glycemic control, the dose may be increased up to the maximum of 25 mg daily. In patients with T2DM, the glycemic efficacy of empagliflozin is dependent on renal function because of its mechanism of action—its efficacy is reduced in patients with moderate renal impairment and likely absent in case of severe renal impairment. If further control of glycemia is needed in patients with T2DM and coexisting renal impairment, the addition of other anti-hyperglycemic agents should be considered. Empagliflozin is not recommended if eGFR is less than 30 mL/min/1.73 m^2^ [11].

## 2. Empagliflozin

### 2.1. Mechanism of Action

The liver, muscles, adipocytes, pancreas and neuroendocrine system play a role in maintaining normal glucose homeostasis. Moreover, studies have revealed that by reabsorbing the filtered glucose, the kidneys also play a central role in the maintenance of glucose homeostasis and ensure the availability of sufficient energy during periods of fasting. In diabetes mellitus (DM), this mechanism becomes maladaptive because of the augmentation of the expression and activity of SGLT2 in the proximal tubule of the kidney caused by hyperglycemia [12].

SGLT2, located in the proximal convoluted segment of the proximal tubule, is a high-capacity, low-affinity glucose transporter. It integrates glucose transport to the electrochemical Na^+^ gradient and mediates 90% of renal glucose reabsorption, while the remaining 10% is reabsorbed by the high-affinity, low-capacity SGLT1 transporter. The mechanism of SGLT2 is that the sodium absorbed across the luminal cell membrane creates a gradient that permits the passive entry of glucose into the cell. Afterwards, the sodium is returned to the bloodstream by the adenosine triphoshatase-mediated sodium–potassium pump and glucose diffuses to the basolateral glucose transporter 2, through which it passes back into the bloodstream [12].

Hyperglycemia augments the expression and activity of the SGLT2, which can cause an increase in glucose reabsorption by up to 20% in individuals with poorly controlled DM. Empagliflozin inhibits SGLT2 and therefore reduces the renal reabsorption of filtered glucose and lowers the renal tubular threshold for glucosuria. This results in increased urinary glucose excretion and reduction in hyperglycemia [5]. Its action leads to excretion of 60–100 g of glucose a day, improvement of glucose control with low risk of hypoglycemia and also result in loss of 240–400 kcal/day into the urine with associated weight reduction [13,14]. In healthy volunteers, empagliflozin produces a dose-dependent increase in urinary glucose excretion of up to 90 g per day [5].

Moreover, empagliflozin is proven to exert many other favorable effects in patients with T2DM such as weight loss, reduction in blood pressure (BP) and serum uric acid levels [14].

### 2.2. Indication for Administration

The principal indication for empagliflozin is T2DM [2]. It is recommended for the treatment of adults with inadequately controlled T2DM as an adjunct to diet and exercise. It can also be included as an adjunct to other hypoglycemic drugs or when metformin is not tolerated [11]. Empagliflozin is the first antidiabetic drug that demonstrated a reduction in mortality in the treatment of T2DM [15]. The agent has been shown to lower mean fasting glucose [16], postprandial glucose [17], and when used in monotherapy or with other hypoglycemic drugs, it lowers HbA1c in patients with T2DM [16]. In addition, empagliflozin has been shown to improve pancreatic beta-cell function by reducing pancreatic beta-cell burden and glucotoxicity [17]. The EMPA-REG OUTCOME [6], EMPEROR-Reduced [18,19] and EMPEROR-Preserved [20] studies, which will be described in detail in the following paragraphs, have shown that there are benefits of empagliflozin in heart failure (HF), both and patients with and without T2DM [6,18,19,20]. The EMPEROR-Reduced trial reported that in HF patients with reduced ejection fraction (HFrEF), empagliflozin slowed the deterioration of renal function, regardless of the presence of T2DM [18]. In addition, its use reduced HFrEF hospitalizations as well as cardiovascular deaths [18,19]. Similar conclusions regarding the effect of empagliflozin on cardiovascular disease (CVD), including HF, came from the EMPEROR-Preserved study involving patients with HF with preserved ejection fraction (HFpEF) [20,21]. According to the 2021 European Society of Cardiology (ESC) guidelines for HF, empagliflozin, along with other SGLT2 inhibitors, is in Class I recommendations for patients with HFrEF, T2DM and HF, T2DM with high-risk CV disease, and T2DM with HFrEF [22].

### 2.3. Side Effects and Contraindications

Despite its many advantages, empagliflozin also has several side effects [23]. Elderly patients with low BP, impaired renal function or using diuretics with empagliflozin are at risk of symptomatic hypotension, since empagliflozin, while exhibiting a diuretic effect, reduces intravascular volume [16,23]. A noteworthy potential complication assessed by Imprialos et al. [24] is the risk of stroke during SGLT2 inhibitor therapy. The occurrence of this complication appears to be a paradox, as the drug originally showed cardiovascular risk-reducing properties including the risk of stroke. The factor responsible for this condition could be an increase in hematocrit and blood viscosity, both of which are effects of pharmacotherapy; however, further studies are needed, as the study that showed this did not reach statistical significance [24]. It is also essential to assess and then monitor renal function before including empagliflozin, as it lowers eGFR and raises serum creatinine levels [23]. It may contribute to the development of a severe but rare complication, ketoacidosis, so it should not be included in patients susceptible to this abnormality [23,25]. The risk of euglycemic ketoacidosis may be increased through the excessive production of ketone bodies by two mechanisms—increasing glucagon secretion or increasing glucosuria and thereby decreasing insulin secretion, resulting in an increased production of free fatty acids (FFA) [26]. Alcohol-dependent patients and those with pancreatic disease are particularly burdened with the occurrence of ketoacidosis [23]. Empagliflozin also exacerbates hypoglycemia in patients taking potent glycemic-lowering treatments such as insulin and insulin-secreting drugs [16,23]. Inducing glucosuria can lead to the development of particularly burdensome fungal infections—genital candidiasis and urinary tract infection, which is mostly a mild infection, but in extreme cases, it can even cause sepsis and death [23,25,27]. Women in particular are at risk for both conditions [23,25]. Fournier’s gangrene, which requires surgical debridement of the tissues, can also be a serious complication [23].

In one study in which empagliflozin was added to metformin treatment, the most commonly reported side effects were urinary tract infection and nasopharyngitis. In addition, episodes of symptomatic hypoglycemia with glucose levels between 3.0 and 3.9 mmol/L were also observed in this group of patients [28]. However, in the EMPA-REG OUTCOME study, it was noted that the incidence of hypoglycemia increased with age regardless of the treatment used without assuming a higher frequency in patients taking empagliflozin. In the same group, a decrease in glomerular filtration rate (GFR) was observed only at the beginning of the study, after which it remained stable, compared to the placebo group, in which GFR levels successively decreased. It was also noted that the frequency of episodes of hypotension was higher in the group using empagliflozin but only in the group of patients 75 years and older. A significant proportion of empagliflozin adverse events are associated with conditions that progress with age, and it is these patients who most often use the drug described [29]. Such a link has been observed with regard to the risk of bone fractures, and empagliflozin has not been shown to increase this risk [26,29]. A similar situation applies to acute kidney injury or dehydration (which, despite not using the drug, also increase with age) [29]. In a study conducted by Donnan et al. [26], SGLT2 inhibitors, regardless of the drug used, compared to placebo and other hypoglycemic drugs, showed no increased risk of developing urinary tract infections. However, when drugs in this group were compared with each other, dapagliflozin had a higher risk of this complication [26]. Table 1 shows some of the side effects of empagliflozin [23,30,31].

Empagliflozin should not be used in patients with type 1 diabetes mellitus (T1DM), those with severe hypersensitivity reactions such as angioedema or anaphylaxis, patients with ketoacidosis, those with end-stage renal disease and those on dialysis [23,30]. It is contraindicated to include the drug when GFR < 30 mL/min/1.73 m^2^, while when it is less than 45 mL/min/1.73 m^2^, its use is not recommended. It is also not recommended to use the drug during the 2nd and 3rd trimesters of pregnancy and in a group of people over 85 years of age [23,25]. A unique group of patients are women of childbearing age, in whom the safety of SGLT2 inhibitors is uncertain. Sotagliflozin is a drug used in patients with T1DM, much of it in women of childbearing age. On the one hand, it has a desirable hypoglycemic effect, but on the other hand, if pregnancy occurs, it can promote ketoacidosis and ultimately pose a risk of fetal loss [32]. It is suspected that empagliflozin crossing the placenta may impair fetal renal development in pregnant women, and it may cause serious side effects in lactating women [16].

## 3. Impact of Empagliflozin in Patients with Chronic Kidney Disease

Chronic kidney disease (CKD) is becoming increasingly one of the major burdens to health care, and currently, the incidence of its occurrence in the population is 10% and is constantly growing [33]. The main hallmarks of CKD are reduced GFR and increased urinary albumin excretion. Reasons for the occurrence of CKD are complex and include common diseases such as metabolic syndrome, DM, obesity or hypertension. It was also observed that the risk of kidney disease has a significant genetic origin [34].

**Figure 2:**
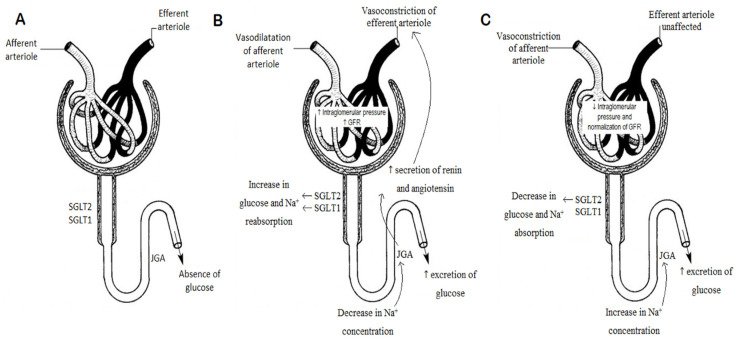
(**A**) Properly functioning nephron. (**B**) The mechanism of nephron impairment in the course of long-term, inappropriately treated DM. (**C**) Effect of SGLT2 inhibitor empagliflozin on improving intraglomerular pressure and GFR in DM [42]. SGLT1, SGLT2—sodium-glucose transport proteins, JGA—juxtaglomerular apparatus.

One of the most common reasons for kidney disease is long-term and inadequately treated DM [35,36]. The progression of kidney disease in DM is divided into early (hemodynamic and metabolic) and late (cell and tissue remodeling) stage, and it is related to the pathophysiological events [37,38,39]. The tubular glucose reabsorption capacity (Tmg) increases with greater glucose re-uptake, which entails increased sodium resorption. Reduced sodium concentration reaches the macula densa and, via tubuloglomerular feedback, activation of the renin–angiotensin–aldosterone system occurs. Consequently, the vasoconstriction of efferent arterioles and vasodilatation of afferent arterioles occur, generating an increase in intraglomerular pressure and GFR (Figure 2) [37,40]. Over time, the continuous increase in pressure and hyperfiltration leads to glomerular damage and the onset of symptoms characteristic of diabetes-induced kidney disease—albuminuria and hypertension [7,41].

Renal improvement is possible with SGLT2 inhibitors, among which is empagliflozin. Its nephroprotective effect is based on both a reduction in the threshold for glucosuria and Tmg. The effect is a reversible inhibition of the SGLT2 receptor, which leads to increased glucose excretion from the body, reduced hyperfiltration and, consequently, intraglomerular pressure [43]. In addition, it lowers renal energy requirements, thereby reducing renal hypoxia. It has antioxidant and anti-inflammatory effects due to a reduction in the expression of inflammatory molecules [4,42]. Thus, empagliflozin has a beneficial effect on improving the basic elements of pathophysiology involved in the development of diabetes-induced kidney disease [8].

The EMPA-REG OUTCOME clinical trial was designed to focus entirely on empagliflozin in people with T2DM with coexisting CVD and an estimated glomerular filtration rate of at least 30 mL/min/1.73 m^2^. This population was specifically examined for the occurrence of clinically significant renal events, i.e., progression to macroalbuminuria, doubling of serum creatinine levels, initiation of renal replacement therapy (RRT), death resulting from renal failure and incident albuminuria [44]. Consequently, the use of empagliflozin in patients resulted in a significant relative risk reduction in developing or worsening nephropathy of 39% compared to the placebo group [45]. Similarly, for progression to macroalbuminuria, the relative risk reduction between the empagliflozin and placebo groups was 38%. The risk of doubling plasma creatinine levels was 44% reduced in the empagliflozin group, and the relative risk of starting RRT was significantly 55% lower. In contrast, there was no significant difference between the groups in the incidence of incidental albuminuria and the occurrence of death caused by renal failure [44,46,47].

Summarizing, patients treated with empagliflozin did not experience progression of CKD, and there was a decrease in the incidence of clinically significant renal events. It was observed that in both groups treated with empagliflozin, there was a decrease in eGFR of 4 mL/min/1.73 m^2^ in the first weeks, which was associated with the tubuloglomerular feedback mechanism described earlier in the chapter. For the remainder of the study, the eGFR value remained stable in contrast to the placebo group [37,48].

In a subsequent study by Cherney et al. [49], a pooled analysis of patients with CKD was performed on the effect of empagliflozin on the urinary albumin-to-creatinine ratio (UACR). They noted that after 24 weeks of empagliflozin administration, UACR values decreased significantly compared to the placebo group. In the microalbuminuria group, the difference was −32%, whereas in the macroalbuminuria group, it was −41%. This difference was significant after the first 12 weeks of treatment, and the effect persisted for the remainder of the study [49]. In addition, the study focused on changes in HbA_1C_, patients’ body weight and BP, which are strongly associated with the occurrence of albuminuria in patients with T2DM [50]. It turned out that the use of empagliflozin resulted in improvements in these variables as well, of which changes in BP had a statistically significant effect on reducing albuminuria in patients with micro- and macroalbuminuria, while change in body weight was significant only in patients with microalbuminuria [49].

Another study conducted, EMPA-REG RENAL, was designed to investigate the improvement of HbA_1C_ in patients with T2DM and concomitant CKD. Patients were classified into groups according to the stage of renal failure (stage 2 with eGFR ≥ 60 and <90 mL/min/1.73 m^2^, stage 3 with eGFR ≥30 and <60 mL/min/1.73 m^2^, and stage 4 with eGFR ≥ 15 and <30 mL/min/1.73 m^2^). The study was also concerned with renal parameters, meaning changes in eGFR and UACR. A slight reduction in eGFR was observed in stage 2, 3 or 4 patients taking empagliflozin, which returned to baseline values at week 3 after the end of treatment. Over the course of the study, patients taking empagliflozin improved UACR compared to placebo. The adjusted mean difference in patients with stage 2 CKD was −184.59, *p* = 0.0831 and −235.86, *p* = 0.0257 for empagliflozin 10 mg and 25 mg, respectively, and −183.78, *p* = 0.0031 for patients with stage 3 CKD. Furthermore, by the end of empagliflozin treatment, the majority of patients with stage 3 CKD had improved baseline albuminuria from initial macroalbuminuria to microalbuminuria (32.6% vs. 8.6% relative to placebo) and initial microalbuminuria to no albuminuria (27.5% vs. 21.4% compared to placebo) [51].

The clinical trial conducted by Zannad et al. [18], which was based on the EMPEROR-Reduced trial, was designed to assess renal outcomes in patients with CKD and HF [18]. In addition, the direct effects of empagliflozin on the kidneys were examined using a prespecified composite renal outcome defined as sustained profound decline in eGFR, chronic dialysis or transplantation [52]. Patients were randomized by the presence or absence of CKD (defined as eGFR < 60 mL/min/1.73 m^2^ or UACR > 300 mg/g) at baseline. Empagliflozin use in patients with CKD reduced the decrease in eGFR by 1.11 mL/min/1.73 m^2^ per year and by 2.41 mL/min/1.73 m^2^ per year in patients without CKD, whereas in the placebo group, eGFR gradually decreased throughout the study. The risk of composite renal outcome was also reduced in patients with and without CKD by HR = 0.53 (95% CI 0.31–0.91) and HR = 0.46 (95% CI 0.22–0.99), respectively [18]. All of the studies described above are included in Table 2 [18,44,49,51].

Moreover, studies conducted with KDIGO (the Kidney Disease Improving Global Outcomes) risk categories show that in both the placebo and empagliflozin groups, the frequency of adverse events was similar. The exception was genital infections occurring more frequently in patients receiving empagliflozin. In contrast, cardiovascular and renal outcomes were consistent across all KDIGO risk categories, demonstrating that regardless of baseline CKD status, treatment with empagliflozin has beneficial effects [53].

Throughout the years, other studies have also appeared to evaluate renal parameters in patients receiving empagliflozin. The drug has proven effective not only in people with CKD but also CVD [52]. Studies conducted in this direction measured changes in GFR and albuminuria in patients with coexisting HF and CKD. The result was a significant reduction in the risk of end-stage renal failure, death from renal causes as well as from cardiovascular causes [54]. Importantly, the latest study analyses confirm the safety of empagliflozin in people with concomitant DM, HF and renal insufficiency at an eGFR of 20 mL/min/1.73 m^2^ [55]. Comparing the other SGLT2 inhibitors with the mentioned data, dapagliflozin should not be administered in patients whose eGFR is below 60 mL/min/1.73 m^2^ [56], in the case of canagliflozin at an eGFR below 45 mL/min/1.73 m^2^. On the other hand, once eGFR values are between 45 and 60 mL/min/1.73 m^2^, its dose should be reduced to 100 mg per day [57].

It is important to keep in mind that as renal function deteriorates, the therapeutic effect of SGLT2 inhibitors, which are based on GFR, diminishes [58,59]. A post hoc analysis of a long-term randomized trial analyzed the differences that occur in patients receiving dapagliflozin in doses of 5 mg and 10 mg versus those on placebo. The intake of dapagliflozin by patients with DM type 2 and stage 3A renal failure was associated with moderate reductions in HbA1c values of −0.33% and −0.37%, respectively, while no change was observed in the stage 3B group compared with the placebo [60]. As for canagliflozin, the use of 100 mg and 300 mg doses in patients at a similar stage of renal failure resulted in a reduction in HbA_1C_ values of −0.27% and −0.41% (95%Cl), respectively [61]. Compared to this, empagliflozin performs much more favorably, as marked changes in HbA_1C_ levels were observed in both stage 2 (the adjusted mean treatment difference between the placebo and empagliflozin 10 mg and 25 mg groups was −0.52% and −0.68%, respectively) and stage 3 (the adjusted mean treatment difference between the placebo and empagliflozin 25 mg groups was 0.42%), and only in stage 4 of renal failure was no improvement observed [51]. 

From the previous paragraphs, we can find out that empagliflozin contributed to the improvement of renal parameters and patients had a reduction in albuminuria. Similar correlations were noted for dapagliflozin and canagliflozin. In a study devoted to dapagliflozin, out of 168 patients receiving 5 mg or 10 mg of SGLT2 inhibitor, there was improvement in 38 patients and progression occurred in 18 compared to the placebo group (comprising 84 patients), where a similar number of patients had improvement or worsening in UACR (9 and 12 patients, respectively) [60]. Canagliflozin administration was also associated with a reduction in the progression of renal failure. In a study of 42 Japanese patients, a reduction in albuminuria was observed in those taking 100 mg of canagliflozin compared to the control group [62].

In studies examining the safety of SGLT2 inhibitors in patients with cardiovascular risk (EMPA-REG OUTCOME, the CANVAS Program, DECLARE-TIMI) [31,44,63,64], some included renal outcomes as secondary endpoints. In the CANVAS Program, 10,142 patients were randomized in two studies (CANVAS and CANVAS Renal Endpoints). The trials showed that patients receiving canagliflozin had a reduced incidence of albuminuria (HR = 1.70; 95% CI, 1.51–1.91), a reduced risk of developing micro- or macroalbuminuria (HR = 0.73; 95% CI, 0.67–0.79), and a less frequent occurrence of the composite outcome of a sustained 40% reduction in eGFR, need for RRT, or death from renal causes (HR = 0.60; 95% CI, 0.47–0.77) [31]. For the DECLARE-TIMI 58 trial, dapagliflozin 10 mg was used in patients, and when analyzing the occurrence of a secondary composite outcome consisting of eGFR decline ≥40%, progression to end-stage renal failure, or death from renal causes, it came out that the risk of the above events was lower in patients receiving dapagliflozin compared to placebo (HR = 0.53 95% Cl, 0.43–0.66) [64].

Another study related to the use of dapagliflozin in patients with renal failure examined two composite outcomes. For the first primary composite outcome of a sustained decline in estimated GFR of at least 50%, end-stage renal disease or death from renal or cardiovascular causes occurred in 197 participants (9.2%) in the dapagliflozin group and 312 participants (14.5%) in the placebo group (HR, –0.61; 95% Cl, 0.51 to 0.72; *p* < 0.001). However, for the second composite outcome (sustained decline in the estimated GFR of at least 50%, end-stage kidney disease, or death from renal causes), dapagliflozin also had better results than placebo 0.56 (95% Cl, 0.45–0.68; *p* < 0.001) [65].

## 4. Impact of Empagliflozin in Patients with Heart Failure

SGLT2 inhibitors were primarily oriented to be the antidiabetic drug with promising results. Initially, they were additional drugs in DM therapy, but during further investigation, results turned out to be far greater than expected. The EMPA-REG OUTCOME trial has provided evidence that SGLT2 inhibitors could become one of the most important drugs in the treatment of HF. This research started rapid growth in exploration for more possible benefits of this drug group and changed the recommendations for treating DM and HF [66].

**Figure 3:**
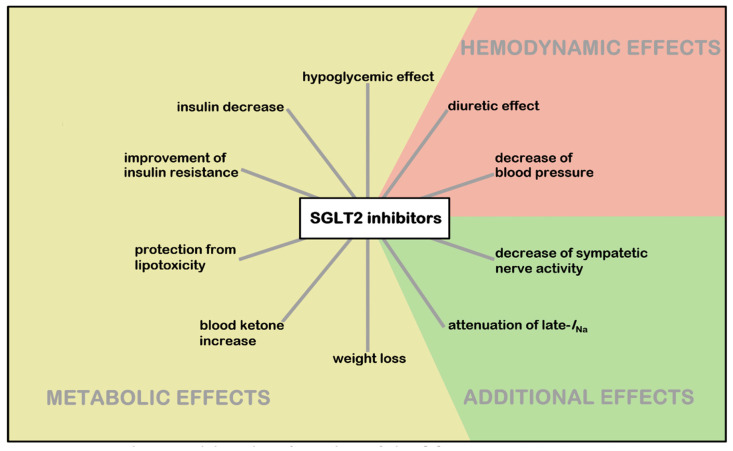
Potential SGLT2 inhibitor benefits in heart failure [67].

The precise mechanism of how SGLT2 inhibitors work in HF is unknown. SGLT2 inhibitors can interact with oxidative stress, inflammation and other factors as exemplified by SGLT2 inhibitors’ influence on the pathophysiology of diabetic cardiomyopathy. Potential mechanisms of these interactions are presented in Figure 3 [67].

Due to the complex mechanisms and their multiplicity, in this paper, we will focus on two factors. The amount of FFA is increased because of DM and obesity; its excessive amounts are causing changes in the cellular metabolism of cardiac muscle cells. Metabolism is converted to using mainly FFA which leads to mitochondrial dysfunction, increased oxidative stress and exacerbation of insulin resistance. Fat accumulation also results in impaired glucose tolerance, dyslipidemia and hypertension due to fat accumulation in the liver and skeletal muscles. These factors are probably involved in stiffening and decreasing myocardial performance. Another possible mechanism of SGLT2 inhibitors is to reduce oxidative stress in the cellular matrix and blood vessels. Excessive ROS production leads to myocardial cell damage, including mitochondrial dysfunction, microvascular dysfunction or cardiomyocyte Ca^2+^ dyshomeostasis. This results in cardiac cell death, fibrosis, and contractile dysfunction, which ultimately leads to HF [67,68,69]. Recently, there have been a few studies attempting to investigate how SGLT2 inhibitors work, such as the study by Kolijn et al. [70] in which authors conduct research on human HFpEF myocardium and obese rats with ZDF. As a result, the authors showed that empagliflozin reduced oxidative stress and myocardial inflammation, improved endothelial function and thanks to that, pathological suppression of the NO–sGC–cGMP–PKG pathway and its downstream targets became reversed, resulting in reduced pathological cardiomyocyte stiffness [70]. Another example of investigation on SGLT2 inhibitor mechanisms is the research conducted by Chenguang Li et al. [71] The aim of their study was to investigate the effect of empagliflozin on myocardium injury and the potential mechanism in type 2 diabetic KK-Ay mice. Results showed that inhibition of the transforming growth factor β/Smad pathway and activation of Nrf2/ARE signaling are the outcomes of empagliflozin suppressing the oxidative stress and fibrosis [71].

Cardiovascular outcomes in EMPA-REG OUTCOME were very promising for patients with HF. Study results showed a significant reduction in hospitalization for HF by 35%. Moreover, the risk of death from any cause and death from cardiovascular causes were 32% and 38% lower in the empagliflozin group, respectively. The primary outcome, that is, death from cardiovascular causes, nonfatal myocardial infarction (MI) or nonfatal stroke, was about 14% less in the empagliflozin group. No relevant differences were noticed between the groups in the incidence of myocardial infarction or stroke. This study was very important and fundamental for future HF treatment guidelines changes due to its strong evidence for a reduction in cardiovascular risk [63,72,73].

The subsequent studies were EMPEROR-Reduced and EMPEROR-Preserved, which contributed much to the knowledge of empagliflozin. The first targeted patients with reduced left ventricle ejection fraction (LVEF), while the second complementarily targeted patients with preserved LVEF. The EMPEROR-Reduced trial confirmed a reduction in the combined risk of death, hospitalization for HF or an emergent/urgent HF visit requiring intravenous treatment (empagliflozin vs. placebo, HR, 0.76; 95% CI, 0.67–0.87; *p* < 0.0001) after adding empagliflozin to standard therapy of HF with reduced EF, and at the 12th day after randomization, those results became statistically significant. The aforementioned reduction was consistent in both diabetic or non-diabetic patients with reduced LVEF. However, this is not the only result that became statistically significant in the first month of the trial. Improvements in New York Heart Association (NYHA) class were statistically significant 28 days after randomization and remained significant during the long-term follow-up. What is important is that lowering the risk of hospital admission was not counterbalanced by prolonged hospitalization in the empagliflozin group. This study together with the DAPA-HF trial supported the position of SGLT2 inhibitors as part of standard of care for patients with HF and a reduced ejection fraction (EF) with or without DM [19,52,74,75].

Patients with preserved LVEF were enrolled in the EMPEROR-Preserved trial to test whether empagliflozin is effective in higher EF and, in a subsequent analysis, whether the presence of DM affects the outcome of patients with HFpEF. The reduction in the combined risk of death from cardiovascular causes, hospitalization for HF, or an emergency or urgent visit for HF requiring intravenous treatment was 23%, making it similar to the results of the EMPEROR-Reduced trial. After 18 days of therapy with empagliflozin, the benefits reached statistical significance and remained significant thereafter. The benefit in the total number of HF hospitalizations was similar in patients with a diastolic EF of between 40% and 50% and between 50% and 60%, while it was reduced at higher EF values. Patients in the empagliflozin group after receiving SGLT2 inhibitor were 20% to 50% more likely to have better NYHA class than placebo patients. What is new in this study is that patients with DM were younger, were more frequently men, and had higher body mass index (BMI) and HbA1c levels. Diabetic patients also had a higher predisposition for ischemic heart disease as the main cause of HF and a higher prevalence of hypertension and coronary artery disease. Furthermore, patients with DM had a worse NYHA class but at the same time lower levels of N-terminal pro b-type natriuretic peptide (NT-proBNP) than those without DM, which may be explained by the association of obesity with lower natriuretic peptide levels and by the lower prevalence of atrial fibrillation (AF) in diabetic patients compared with those without DM. Since obesity is associated with lower natriuretic peptide levels, patients having lower median NT-proBNP values despite a worse NYHA class could be partly explained by a higher BMI. This study also shows that patients with prediabetes and normoglycemic have lower occurrence of primary end point and first and recurrent hospitalization for HF than those with DM. The rates between the prediabetes and normoglycemic were similar. The prevalence of DM in HFpEF is confirmed by clinical studies and registries. One of them is the EMPEROR-Preserved Trial in which 49% of the patients enrolled had DM, 33% had prediabetes and only 18% of the total trial population had normoglycemia [20,75,76].

In both the EMPEROR-Reduced and EMPEROR-Preserved trials, empagliflozin reduced the risk of HF outcomes irrespective of diabetes status at baseline. Summarizing the results and data from these two studies, the reduction in both time to first HF hospitalization and total (first and recurrent) HF hospitalization was similar (by 25–35%) in the range of <25% to <65% of patients’ EF. Moreover, in those studies, sex has no proven influence on EF [74,75,76].

Although the above-mentioned studies have significant implications for the better treatment of HF, there are many other researchers contributing to a better understanding of SGLT2 inhibitors. EMPULSE was another research study on empagliflozin. This study included patients with acute HF with no prior history of HF. There had been previous attempts to study this group, but they were unsuccessful because SGLT2 inhibitor was administered too briefly (24 or 48 h). There were also concerns about the safety of starting SGLT2 inhibitor treatment so early. Reassuringly, the study ended with positive effects showing that adding empagliflozin to standard therapy in the early stage is safe and well tolerated with fewer serious adverse events than placebo. Moreover, this modification of acute HF therapy granted clinical benefits defined as a hierarchical composite of death, time to first HF event number of HF events or change from baseline in the Kansas City Cardiomyopathy Questionnaire Total Symptom Score compared with placebo (stratified win ratio 1.36, 95% CI 1.09–1.68; *p* = 0.0054) [77,78].

EMPA-TROPISM investigated how empagliflozin influences left ventricle (LV) properties. That was the first study which demonstrated that empagliflozin ameliorates LV remodeling in nondiabetic HF patients. Santos-Gallego et al. [79] used cardiovascular magnetic resonance (CMR) to describe that empagliflozin reduces LV volume, mitigates LV hypertrophy, makes LV less spherical, and increases LVEF. The authors of the study highlighted that the reductions in both left ventricular end-diastolic volume (LVEDV; −25.1 ± 26.0 mL vs. −1.5 ± 25.4 mL for empagliflozin vs. placebo; *p* < 0.001) and left ventricular end-systolic volume (LVESV; −26.6 ± 20.5 mL vs. −0.5 ± 21.9 mL for empagliflozin vs. placebo; *p* < 0.001) were significant in the empagliflozin group. Moreover, the regression of LV hypertrophy, LV mass reduction (−17.8 ± 31.9 g vs. 4.1 ± 13.4 g for empagliflozin vs. placebo; *p* < 0.001) and LVEF increase (6.0 ± 4.2 vs. −0.1 ± 3.9 for empagliflozin vs. placebo; *p* < 0.001) were significant during empagliflozin treatment [79].

The SUGAR-DM-HF study, which had the same objective as EMPA-TROPISM and imagining technique (CMR), also had similar results. SUGAR-DM-HF confirmed that empagliflozin can improve LV parameters. The authors reported a reduction in left ventricle (LV) end-systolic volume index by 6.0 (95% CI, −10.8 to −1.2) mL/m^2^ (*p* = 0.015) and LV end-diastolic volume index by 8.2 (95% CI, −13.7 to −2.6) mL/m^2^ (*p* = 0.0042) [80].

In the EMPULSE Trial, patients were assessed four times by using the Kansas City Cardiomyopathy Questionnaire (KCCQ) at randomization and at 15, 30 and 90 days, which was the length of this trial. This study showed that the beneficial effects of empagliflozin on HF outcomes in this patient group are independent of the health status impairment at baseline. The empagliflozin group had greater improvement in all key KCCQ domains, including Total Symptom Score (TSS), physical limitations (PLS), quality of life (QoL), clinical summary score (CSS), and overall summary score (OSS) at last assess (placebo-adjusted mean differences [95% CI]: 4.45 [95% CI, 0.32–8.59], *p* = 0.03; 4.80 [95% CI, 0.00–9.61], *p* = 0.05; 4.66 [95% CI, 0.32–9.01], *p* = 0.04; 4.85 [95% CI, 0.77–8.92], *p* = 0.02; and 4.40 [95% CI, 0.33–8.48], *p* = 0.03, respectively), and this improvement was present from the 15th day until the end of the trial. Patients were enrolled and evaluated while they were still in the hospital, so patients with de novo acute HF or decompensated chronic HF were also included. Importantly, improvements in the symptoms and functional status in the early post-discharge period in hospitalized patients with acute HF have been previously demonstrated with only a few treatments. KCCQ is known as a predictor of cardiovascular death and HF readmission. Better KCCQ scores in the empagliflozin group showed that the rapid implementation of SGLT2 inhibitors could be beneficial to patients in the postdischarge period. Since that was the first such observation, we need to wait for future studies [81].

Since the publication of EMPA-REG OUTCOME results, there have been additions to the SGLT2 inhibitor family. Until now, we have examined five SGLT2 inhibitors in terms of HF: empagliflozin, dapagliflozin, canagliflozin, ertugliflozin and the most recent sotagliflozin.

Dapagliflozin in the DECLARE-TIMI 58 study did not significantly reduce the primary composite outcome including MACE (HR, 0.93; 95% CI, 0.84–1.03; *p* = 0.17) and hospitalization, but on the other hand, it did result in decreasing the rate of HFH or cardiovascular death by 17% (HR, 0.83; 95% CI, 0.75–0.95). It was observed that a reduction in the number of cardiovascular deaths or hospitalizations is more visible in patients with HFrEF (HR 0.62, 95% CI, 0.45–0.86) in comparison to those with HFpEF (HR, 0.88; 95% CI, 0.76–1.02; *p*-interaction 0.046) [64,82,83]. In comparison, the DAPA-HF study dapagliflozin reduced the primary outcome (composite of worsening HF (hospitalization or an urgent visit resulting in intravenous therapy for HF) or cardiovascular death) occurrence (HR, 0.74; 95% CI, 0.65 to 0.85; *p* < 0.001) and significantly decreased cardiovascular mortality (HR, 0.82; 95% CI, 0.69–0.98) in patients with HFrEF. A first worsening HF event occurred less often in the empagliflozin group (HF, 0.70; 95% CI, 0.59 to 0.83), and patients less often died from any cause (HR, 0.83; 95% CI, 0.71 to 0.97) [84]. On the other hand, the PRESERVED-HF trial confirmed via using KCCQ that patients with HFpEF also benefit from taking dapagliflozin. Dapagliflozin improved the KCCQ by 5.8 points compared to the placebo group (*p* = 0.001) [85].

Another known SGLT2 inhibitor is canagliflozin. This medication was evaluated in the CANVAS trail. In this study, 10,142 diabetic patients with or without ASCVD took part with a mean follow-up of 188.2 weeks. The primary endpoint (composite of cardiovascular death, nonfatal myocardial infarction, or nonfatal stroke) was reduced with canagliflozin compared with placebo (26.9 versus 31.5/1000 patient-years; HR, 0.86; 95% CI, 0.75–0.97; *p* < 0.001 for noninferiority, *p* = 0.02 for superiority). The outcomes of the CANVAS study were similar to those from DAPA-HF; that is, canagliflozin also reduced heart failure hospitalization (HFH) (HR, 0.67; 95% CI, 0.52–0.87). In addition, patients with a history of HF have a greater reduction in cardiovascular death or HFH (HR, 0.61; 95%, CI, 0.46–0.80) than patients without a history of HF (HR, 0.87, 95% CI, 0.72–1.06; *p* for interaction 0.021). When it comes to adverse reaction, canagliflozin has an increased risk of amputation (6.3 vs. 3.4 participants per 1000 patient-years; HR, 1.97; 95% CI, 1.41 to 2.75) primarily at the level of the toe or metatarsal [31,82,86,87].

One of the newer SGLT2 inhibitors is ertugliflozin. What is important in the results of the VERTIS-CV trial is that ertugliflozin reduced the risk for the first HHF (HR, 0.70 [95% CI, 0.54–0.90]; *p* = 0.006) and that in the overall population, the risk reduction tended to be greater for those with EF ≤ 45% (HR, 0.48; 95% CI, 0.30–0.76) versus EF > 45% (HR, 0.86; 95% CI, 0.58–1.29). In patients with T2DM, ertugliflozin reduced the risk for the first and total HHF, and the total HHF/CV death has been reduced by ertugliflozin in patients with T2DM. The authors also drew attention to the result that amputations were performed more often in the ertugliflozin group: that is, in 54 patients (2.0%) who received the 5 mg dose of ertugliflozin and in 57 patients (2.1%) who received the 15 mg dose as compared with 45 patients (1.6%) who received placebo [82,88,89]. 

The last one and the newest is sotagliflozin, the SGLT1 and SGLT2 inhibitor. Both SCORED and SOLOIST-WHF studies were terminated early because of the loss of funding [82]. In the SCORED study, the primary end point was changed during the trial and redefined as the composite outcome of CV death, HFH, and urgent visits for HF. The primary end point has lesser occurrence in the sotagliflozin group than in the placebo group (HR, 0.74; 95% CI, 0.63–0.88). The first occurrence of death from cardiovascular causes, nonfatal myocardial infarction, or nonfatal stroke and the first occurrence of death from cardiovascular causes or hospitalization for heart failure were also in favor for sotagliflozin, and it was (HR, 0.84; 95% CI, 0.72 to 0.99) and (HR, 0.77;95% CI, 0.66 to 0.91) respectively. Sotagliflozin was neutral compared with placebo in terms of mortality from CV causes (HR, 0.90; 95% CI, 0.73–1.12; *p* = 0.35) [90]. Simultaneously to the SCORED Trial, the SOLOIST-WHF Trial was conducted. The primary end point for this study was the total number of deaths from cardiovascular causes and hospitalizations and urgent visits for heart failure (first and subsequent events). In total, 1222 patients underwent randomization (608 to the sotagliflozin group and 614 to the placebo group) and the primary end point events occurred 600 times in the sotagliflozin group 245 times and 355 times in the placebo group (HR, 0.67; 95% CI, 0.52 to 0.85; *p* < 0.001). The results of the analysis of the first secondary end point (the total number of hospitalizations and urgent visits for heart failure) were consistent with the results of the primary end-point analysis (HR, 0.64; 95% Cl, 0.49 to 0.83; *p* < 0.001). In this trial, the rate of death from cardiovascular causes (secondary end point) was nonsignificant with 10.6% in the sotagliflozin group and 12.5% in the placebo group (HR, 0.84; 95% CI, 0.58 to 1.22, *p* = 0.36). Other secondary endpoints had a nonsignificant *p* value [91].

In SCORED Trial diarrhea, genital mycotic infections, volume depletion, and diabetic ketoacidosis were more common with sotagliflozin than with placebo, and it was (8.5% vs. 6.0%; *p* < 0.001) for diarrhea, (2.4% vs. 0.9%; *p* < 0.001) for genital mycotic infections, (5.3% vs. 4.0%; *p* = 0.003) for volume depletion and (0.6% vs. 0.3%; *p* = 0.02) for diabetic ketoacidosis. In comparison, the results of the SOLOIST-WHT Trial diarrhea were more common in the sotagliflozin group than in the placebo group (6.1% vs. 3.4%), as was severe hypoglycemia (1.5% vs. 0.3%) [82,90,91].

## 5. Efficiency and Safety in Clinical Trials in Patients with Diabetes Mellitus

Phase 2 and 3 clinical trials have confirmed that empagliflozin improves glycemic control in monotherapy in patients with T2DM. A dose-dependent clinically meaningful reduction in HbA1c levels was achieved as well as reduction in fasting glucose levels and body mass reduction compared to placebo [92].

In a study performed by Rosenstock at al., empagliflozin caused an improvement of glycemic control and weight reduction in obese patients with T2DM with inadequate control on high multiple dose insulin without increasing the risk of hypoglycemia and with lower insulin requirements [93].

A randomized, double-blind and placebo-controlled trial performed by Haring et al. [94] demonstrated that empagliflozin used as an add-on to metformin therapy improves glycemic control in patients with T2DM (during the 24-week trial, significant reductions in HbA1c levels were achieved compared to placebo, and the proportion of patients reaching an HbA1c level of <7% was higher than in placebo group). It also reduces BP and leads to weight loss. Moreover, empagliflozin treatment led to significant improvements in fasting plasma glucose, mean daily glucose and 2 h postprandial glucose levels compared with placebo with low risk of hypoglycemia [94].

Another paper by Haring et al. [95] investigated the efficacy and tolerability of empagliflozin as an add-on to metformin and sulfonylurea in patients with T2DM. In a 24-week, randomized, double-blind, placebo-controlled trial, both 10 mg and 25 mg doses led to clinically meaningful improvements in glycemic control, body weight and systolic blood pressure (SBP) with a good tolerability and safety profile. No improvement was achieved in terms of diastolic blood pressure (DBP) [95].

A 24-week trial of empagliflozine in comparison to sitagliptin in previously untreated patients with T2DM and insufficient glycemic control was performed by Roden et al. [96] Treatment with empagliflozin 10 mg or 25 mg once daily as monotherapy resulted in significant and clinically meaningful improvements in HbA1c and SBP, with a good tolerability profile. In patients with HbA1c lower than 8.5% at baseline, the reduction with empagliflozin was much the same as reductions with sitagliptin, but in patients with at least 8.5% HbA1c at baseline, reductions with empagliflozin 10 mg and 25 mg were greater than were those with sitagliptin. With weight loss being an additional goal of treatment, empagliflozin provided significant reductions in both bodyweight and waist circumference when compared with placebo or sitagliptin [96].

In a Phase 3 trial of empagliflozin as add-on therapy to pioglitazone or pioglitazone plus metformin in patients with T2DM by Kovacs et al. [97], empagliflozin in doses of 10 mg and 25 mg once daily for 24 weeks significantly improved glycemic control (both in HbA1c and fasting plasma glucose levels) compared with placebo and was well tolerated. The efficacy results were similar between patients with pioglitazone alone as background therapy and patients whose background therapy was pioglitazone plus metformin. Treatment with empagliflozin once again resulted in significant weight reduction [97].

Another study evaluating the efficacy of empagliflozin versus placebo when added in patients with inadequate glycemic control treated with linagliptin and metformin performed by Softeland et al. [98] has shown that after 24 weeks, empagliflozin in doses of 10 mg and 25 mg significantly improved HbA1c by 0.79% and 0.70% respectively, versus placebo, and it also reduced fasting plasma glucose levels [98].

Empagliflozin in doses of 10 mg or 25 mg for 52 weeks as an add-on therapy to liraglutide in Japanese patients with T2DM insufficiently controlled by liraglutide alone in a phase 4 trial by Terauchi et al. [99] led to improvements in glycemic control (reduction in HbA1c and fasting plasma glucose levels), decreased body weight, waist circumference, SBP and DBP as well as reduction in fasting plasma insulin levels [99].

Clinical trials have shown that SGLT2 inhibitors also can reduce cardiovascular risk in patients with T2DM by reducing body weight, SBP and serum uric acid [100]. It is also proven that in patients with T2DM empagliflozin reduces the urine albumin-to-creatinine ratio (ACR) in microalbuminuric as well as microalbuminuric ranges [49].

In a pooled analysis of placebo-controlled clinical trials made by Tuttle et al. [55] in 2022, the use of empagliflozin in patients with T2DM and advanced CKD found no overall differences in rates of serious adverse events, adverse events leading to the discontinuation or events of special interest with empagliflozin treatment versus placebo with an exception of genital infections [55].

Comparing the effects of empagliflozin to other SGLT2 inhibitors in patients with T2DM, it is worth noting the cardiovascular outcomes [101,102,103]. In a retrospective cohort study, Suzuki et al. [101] examined the risk of developing CVD events among T2DM patients without a history of CVD with newly initiated SGLT2 inhibitor therapy, and they noted no significant differences in the risk of developing MI, stroke, AF, HF and angina pectoris between patients on therapy with empagliflozin, dapagliflozin, canagliflozin or other SGLT2 inhibitors (ipragliflozin, tofogliflozin, luseogliflozin) [101]. Similarly, when it came to reducing HF worsening, empagliflozin, dapagliflozin and canagliflozin showed comparable effects [102]. However, a meta-analysis by Jiang et al. [103] found that empagliflozin at a dose of 25 mg was the most effective in reducing cardiovascular events, thus showing better efficacy in this aspect than dapagliflozin and canagliflozin [103]. It seems that SGLT2 inhibitors may be an effective tool in the primary prevention of cardiovascular events in patients with DM [101]. In addition, empagliflozin has been shown to reduce all-cause mortality more than canagliflozin and dapagliflozin [102,103]. Similar conclusions can be drawn when analyzing the effect of SGLT2 inhibitors on cardiovascular mortality—empagliflozin has been shown to be more effective than canagliflozin and dapagliflozin [102]. In the meta-analysis from 2016, Tang et al. [104] proved that neither empagliflozin, canagliflozin nor dapagliflozin was associated with increased risk of all-cause mortality and cardiovascular outcomes when used to treat patients with T2DM. Furthermore, the authors speculated about the protective effect of empagliflozin. When used as an add-on to the T2DM therapy, both empagliflozin and dapagliflozin had excellent efficacy, safety, and tolerability profile as proven by Hussain et al. [105]. There is a need to explore the efficacy and safety of SGLT-2 inhibitors further in a group of diabetic patients with cardiovascular disease and renal impairment.

A summary of studies comparing empagliflozin with other SGLT2 inhibitors is presented in Table 3.

## 6. Conclusions

Empagliflozin is a potent SGLT2 inhibitor. The drug’s mechanism of action based on blocking glucose reabsorption in proximal tubules makes empagliflozin exhibit a number of benefits in addition to glucosuria and normoglycemia. The pleiotropic effect of the drug is, among other things, to reduce body weight or lower BP. Although empagliflozin has many advantages, it also causes several side effects among which the most notable are troublesome genital fungal infections and urinary tract infections, symptomatic hypotension, risk of hypoglycemia when used together with insulin or insulin-secreting drugs, and rare ketoacidosis. In addition to treating T2DM, empagliflozin is indicated for the treatment of HF. The EMPEROR-Reduced and EMPEROR-Preserved trials showed that empagliflozin use reduces HF hospitalizations as well as cardiovascular deaths. The conclusion of the EMPEROR-Reduced study is that empagliflozin should be used in HF patients with HFrEF and CKD. The drug’s beneficial effect on the kidneys is based on deceleration of the rate of decline in renal function, reduced incidence of clinically significant renal events, and the lack of progression of CKD. However, note that it should not be used in patients with impaired renal function with GFR < 30 mL/min/1.73 m^2^.

## Figures and Tables

**Table 1:** Examples of empagliflozin side effects [23,30,31].

Side Effects of Empagliflozin
Fungal infections of the genital tract
Urinary tract infection
Symptomatic hypotension
Ketoacidosis
Upper respiratory tract infection
Dyslipidemia
Acute kidney injury
Hypoglycemia (when empagliflozin is used together with insulin or insulin-secreting drugs)
Fournier’s gangrene
Arthralgia
Amputations (canagliflozin)

**Table 2:** Summary of trials of empagliflozin in patients with T2DM and CKD [18,44,49,51].

Authors	Wanner et al., 2016 [44]				Barnett et al., 2014 [51]				Cherney et al., 2016 [49]	Zannad et al., 2021 [18]
Study design	randomised, double-blind, placebo-controlled trial in patients with DM type 2, established CV disease and eGFR at least 30 mL/min/1.73 m^2^				randomised, double-blind, parallel group, placebo-controlled trial in patients with 2, 3 or 4 stage of CKD and HbA1C 7–10%				pooled analysis of patients with CKD and DM type 2 from phase III of clinical trials	randomised, double-blind, placebo-controlled, parallel-group, event-driven trial
All patients	7020				741				851	3730
Baseline renal function	eGFR < 59 mL/min/1.73 m^2^	Group 1. Placebo 2. Empagliflozin	eGFR > 60 mL/min/1.73 m^2^	Group 1. Placebo 2. Empagliflozin	Patients with chronic kidney disease	Stage 2	Stage 3	Stage 4	No of patients with UACR 30–300 mg/g Empagliflozin 388 Placebo 248 UACR > 300 mg/g Empagliflozin 128 Placebo 87	Patients with CKD: eGFR < 60 mL/min/1.73 m^2^ or UACR > 300 mg/g without CKD: eGFR ≥ 60 mL/min/1.73 m^2^ and UACR ≤ 300 mg/g
		eGFR 1. 48.6 mL/min/1.73 m^2^ 2. 48.4 mL/min/1.73 m^2^		eGFR 1. 82.7 mL/min/1.73 m^2^ 2. 83.1 mL/min/1.73 m^2^		eGFR ≥ 60 mL/min/1.73 m^2^ < 90 mL/min/1.73 m^2^	eGFR ≥ 30 mL/min/1.73 m^2^ < 60 mL/min/1.73 m^2^	eGFR ≥ 15 mL/min/1.73 m^2^ < 30 mL/min/1.73 m^2^		
		No of patients with macro-/microalbuminuria 1. 115/488 2. 223/977		No of patients with macro-/microalbuminuria 1. 145/1569 2. 286/3149		290 patients	374 patients	74 patients		
Duration	3.1 years				52 weeks				24 weeks	16 months
Treatment	Empagliflozin 10 mg, 25 mg, Placebo				In stage 2 of CKD Empagliflozin 10 mg, 25 mg, Placebo In stage 3 and 4 of CKD Empagliflozin 25 mg, Placebo				Empagliflozin 10 mg, 25 mg, Placebo	Empagliflozin 10 mg, Placebo
Renal outcome	Empagliflozin vs. placebo Incident or worsening nephropathy (12.7% vs. 18.8%), progression to macroalbuminuria (11.2% vs. 16.2%), doubling of serum creatinine level (1.5% vs. 2.6%), initiation of RRT (0.3% vs. 0.6%), incident albuminuria (51.5% vs. 51.2%)				Baseline to 52 week change of UACR empagliflozin vs. placebo Stage 2 Empagliflozin 10 mg 184.59; Empagliflozin 25 mg 235.86 Stage 3 Empagliflozin 25 mg 183.78 Macroalbuminuria to microalbuminuria 32.6% vs. 8.6%; Microalbuminuria to no albuminuria 27.5% vs. 21.4%				Change in UACR from baseline to 24 week vs. placebo 30–300 mg/g −32%, *p* < 0.001> 300 mg/g −41%, *p* < 0.001	Reduced slope of eGFR by 1.11 mL/min/1.73 m^2^/year (*p* < 0.013) in patients with CKD and 2.41 L/min/1.73 m^2^ (*p* < 0.001) in patients without CKD; composite renal outcome in patients with CKD HR = 0.53 and without CKD HR = 0.46

DM, diabetes mellitus; CKD, chronic kidney disease; eGFR, estimated glomerular filtration rate; RRT, renal replacement therapy; HbA_1C_, glycated hemoglobinA_1C_; UACR, urinary albumin-to-creatinine ratio; HR, hazard ratio.

**Table 3:** Comparison of empagliflozin with other SGLT2 inhibitors in diabetic patients.

Authors	Suzuki et al. [101]	Täger et al. [102]	Jiang et al. [103]	Tang et al. [104]	Hussain et al. [105]
**Year**	2022	2021	2022	2016	2021
**Study design**	Clinical trial	Meta-Analysis	Meta-Analysis	Meta-Analysis	RCT
**All Patients**	25,315	74,874	70,574	29,859	280
**Patient Category**	Diabetic patients with recently implemented SGLT2 inhibitor therapy	52–69 years old; HbA_1C_ 7.2–9.3%	T2DM patients treated with SGLT2 inhibitors	adults with T2DM	T2DM with HbA_1C_ 7.5–11% with different first line anti-diabetic drugs combination
**Type of treatment**	dapagliflozi, empagliflozin, canagliflozi, other SGLT2 inhibitors (tofogliflozi, ipragliflozin and luseogliflozin)	canagliflozin, dapagliflozin, empagliflozin or ertugliflozin (for at least 24 weeks)	placebo; dapagliflozin 2,5 mg, 5 mg, 10 mg; empagliflozin 10 mg, 25 mg; canagliflozin 100 mg, 300 mg	SGLT2 inhibitor vs. placebo or other anti-diabetic treatment	empagliflozin 10–25 mg vs. dapagliflozin 5–10 mg daily as add on therapy
**Comparison of empagliflozin with other SGLT2 inhibitors**	No significant differences in risk of MI, stroke, AF, HF and AP between patients treated with individual SGLT2 inhibitors.	Empagliflozin, dapagliflozin and canagliflozin similarly reduced HF worsening. Empagliflozin is more effective than canagliflozin and dapagliflozin in reducing all-cause mortality and cardiovascular mortality.	Empagliflozin is more effective than canagliflozin and dapagliflozin in reducing cardiovascular events and all-cause mortality.	Neither empagliflozi, canagliflozin nor dapagliflozin was associated with increased risk of all-cause mortality and CV outcomes. Empagliflozin may have a protective effect.	Comparing with dapagliflozi, empagliflozin caused a more significant improvement in body weight, fasting blood sugar and HbA1c over a period of 12 weeks and caused less adverse effects.

SGLT2 inhibitors, sodium-glucose cotransporter-2 inhibitors; MI, myocardial infarction; AF, atrial fibrillation; HF, heart failure; AP, angina pectoris; CV, cardiovascular; RCT, randomized controlled trial.

## Data Availability

The data used in this article are sourced from materials mentioned in the References section.
